# Chemical Composition, Bioactivity and Safety Aspects of Kuding Tea—From Beverage to Herbal Extract

**DOI:** 10.3390/nu12092796

**Published:** 2020-09-12

**Authors:** Svenja Wüpper, Kai Lüersen, Gerald Rimbach

**Affiliations:** Institute of Human Nutrition and Food Science, University of Kiel, Hermann-Rodewald-Strasse 6, 24118 Kiel, Germany; luersen@foodsci.uni-kiel.de (K.L.); rimbach@foodsci.uni-kiel.de (G.R.)

**Keywords:** kuding tea, kudingcha, *Ilex*, herbal drug, extract, bioactivity, safety

## Abstract

Kuding tea (KT) is a bitter-tasting herbal tea that has been commonly used in traditional Chinese medicine (TCM). The large-leaved Ku-Ding-Cha (Aquifoliaceae) is composed of its representative species *Ilex latifolia* Thunb and *Ilex kudingcha* C.J. Tseng. Because of its potential lipid-lowering, body weight-reducing and blood-glucose-lowering properties, KT has increasingly been recognised for its importance over the past several decades. KT is no longer used only as a beverage, and various extraction methods have been applied to obtain aqueous and ethanolic KT extracts (KTE) or their fractions, which could potentially be used as dietary supplements. The major bioactive components of KT are triterpene saponins and polyphenols, but the composition of KT differs substantially between and among the different KT species. This in turn might affect the physiological effects of KT. KT exhibits antiobesity properties, possibly partly by affecting the intestinal microbiota. In addition, KT may mediate putative antioxidative, anti-inflammatory and anticancer activities. However, there is evidence that high KTE supplementation can adversely affect liver metabolism. The physiological relevance of KT in humans remains rather unclear since the potential health benefits of KT and its constituents reviewed here are mainly derived on the basis of in vitro and animal studies.

## 1. Introduction

**Figure 1:**
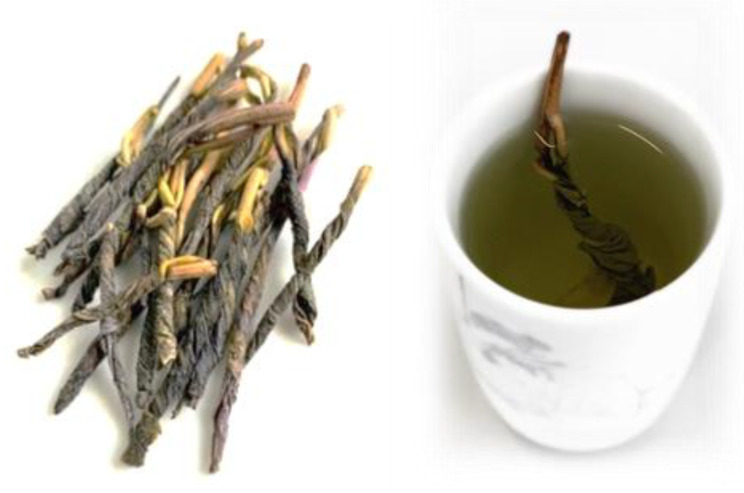
Dried and brewed *Ilex kudingcha* leaves.

Kuding tea (KT, Figure 1) is a bitter-tasting herbal tea that has been commonly used in traditional Chinese medicine (TCM) [1]. Approximately 12 species belonging to different families and genera are collectively called “Ku-Ding-Cha” in different areas of China. The word “Ku” literally means bitter (poor) and “Ding” means man. Both words together also refer to labourers [2]. KT has gained importance and popularity worldwide over the last decade. In particular, its usage as a medicinal plant to counteract heat and toxins in the body and to manage hypertension, hyperlipidaemia and obesity has increasingly been recognised for its importance in recent years [1,2]. The potential health benefits might be attributed to the presence of characteristic phytochemicals such as polyphenols and triterpenoids [1]. KT is no longer used only as a beverage. Various extraction methods have been applied to obtain partly purified extracts of KT. For instance, aqueous extracts, alcoholic extracts and a saponin extract of KT have been examined in vitro and in vivo [3,4,5,6,7]. Moreover, some novel technologies, such as encapsulation or nanotechnology, have been developed to enhance the bioactivity of KT phytochemicals. Kudingcha nanoparticles (KNPs) have been studied for their biological activity [8], and a KT extract encapsulated in γ-cyclodextrin (KTE-γCD) has been examined regarding its effects on liver metabolism [9]. However, if herbal extracts are used as dietary supplements at high concentrations, safety aspects need to be taken into account [9,10].

This review aims to summarise the present state of knowledge regarding the chemical composition, bioactivity and safety aspects of the genus *Ilex* from large-leaved Kudingcha. Some reviews have been published earlier [1,11,12], but these papers did not fully address novel administration approaches of KT or corresponding safety aspects of these new approaches.

## 2. Classification and Distribution

**Figure 2:**
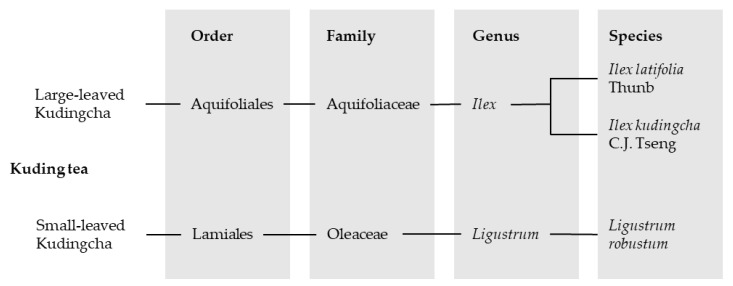
Classification of the most common Kudingcha species consumed in China.

Approximately 12 species belonging to up to six families and six genera are collectively named “Ku-Ding-Cha” [2]. The two most commonly found plant species that are used for the tea can be divided into the groups “large-leaved Ku-Ding-Cha” and “small-leaved Ku-Ding-Cha”. Small-leaved tea belongs to the family Oleaceae and includes species from the genus *Ligustrum*. The large-leaved Kudingcha contains, amongst others, the genus *Ilex* with its representative species *Ilex latifolia* Thunb and *Ilex kudingcha* C.J. Tseng, who have very similar botanical characteristics [1,13]. In addition, there are reports about KT and the species *Ilex kaushue* S.Y. Hu, *Ilex pentagona* S.K. Chen, Y.X. Feng et C.F. Liang and *Ilex cornuta* Lindl. et Paxt., whereby *I. kaushue* appears to be the same species as *I. kudingcha* and *I. pentagona* has been mistakenly classified in the large-leaved Kudingcha group. Presently, *I. cornuta* is known as “Gougucha” and was therefore removed from the KT classification [13]. However, the large-leaved genus *Ilex* from the family Aquifoliaceae was attested to be the original Kudingcha species [1,13] and is, besides *Ligustrum*, the most commonly drank Kudingcha category in China [14]. An overview of the classification of the most popular KT species is given in Figure 2.

**Figure 3:**
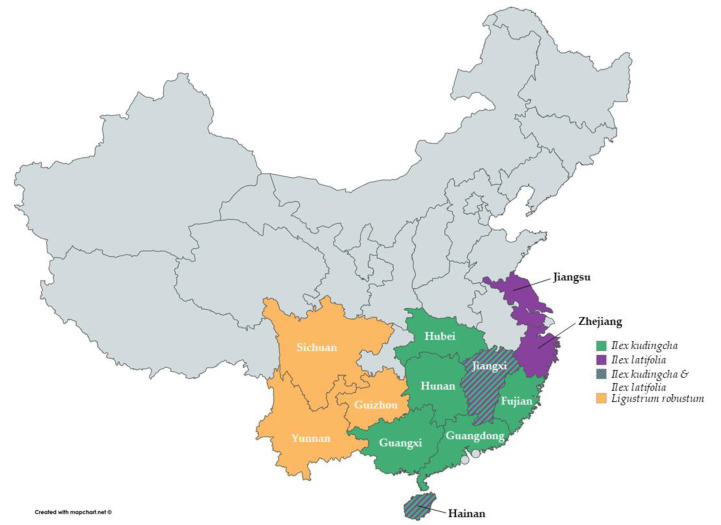
Reported provinces of China where the most common large-leaved Kudingcha species have been mainly found (created with mapchart.net).

The different species of Kudingcha are distributed throughout China across diverse provinces (Figure 3). *I. kudingcha* has been mainly found in the provinces Fujian, Guangxi, Guangdong, Hainan, Hunan, Hubei and Jiangxi [1,13,15], while *I. latifolia* is commonly found in Hainan, Jiangxi, Jiangsu and Zhejiang [1,16,17]. *Ligustrum robustum* has been found in Guizhou, Yunnan and Sichuan [1]. 

## 3. Important Phytochemicals of Large-Leaved Kudingcha

Phenolic acids and triterpenoids are considered to be the major constituents in large-leaved Kudingcha [1,18]. However, it has been shown that the content of these phytochemicals exhibits remarkable differences not only among the various species but also within the same species, even from samples from the same province [13,14,15,19,20,21]. This should be considered when directly comparing different studies on KT. These differences could be attributed to variations in genetics [15], plant origin [15,21], geographical climate [21], age [13], harvest time [21] and environmental factors [15]. Moreover, drying processes [15,21], storage conditions [15] and extraction methods [19,20] may influence the composition of the end product.

### 3.1. Triterpenoids and Their Glycosides

**Figure 4:**
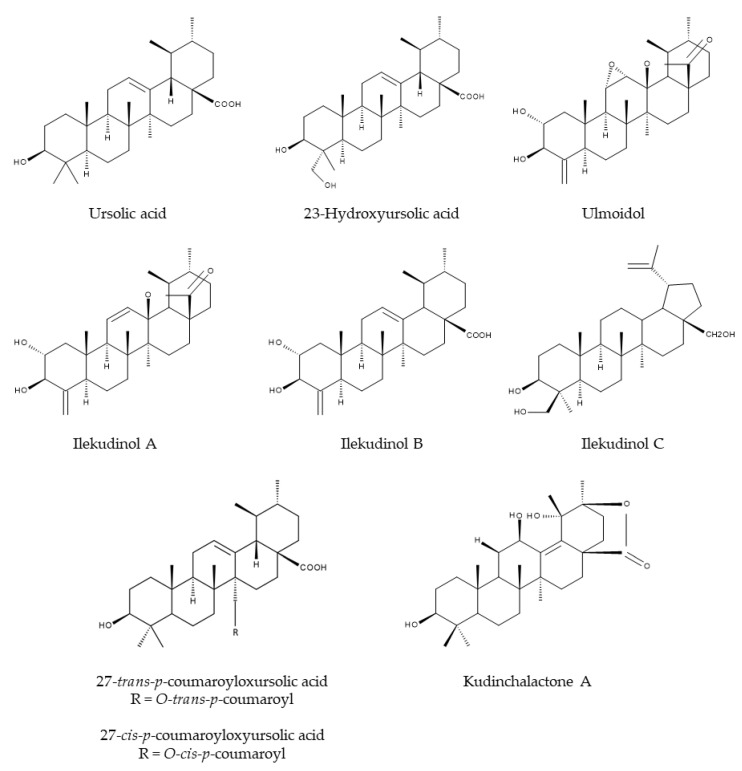
Chemical structures of the triterpenoids in Kuding tea.

In 1996, Ouyang et al. [22] isolated for the first time the two triterpenoids α- and β-kudinlactone from the leaves of *I. kudingcha*. It turned out that α- and β-kudinlactone are ursolic acid (UA) derivatives [22]. Mere UA has also been found in *I. latifolia* [23] and *I. kudingcha* [24]. In addition, many more triterpenoids have been isolated from *Ilex* species, such as ulmoidol [25], 23-hydroxyursolic acid [25], 27-trans-p-coumaroyloxyursolic acid [25], 27-cis-p-coumaroyloxyursolic acid [25], ilekudinols A-C [25] and kudinchalactone A [26] (Figure 4).

**Figure 5:**
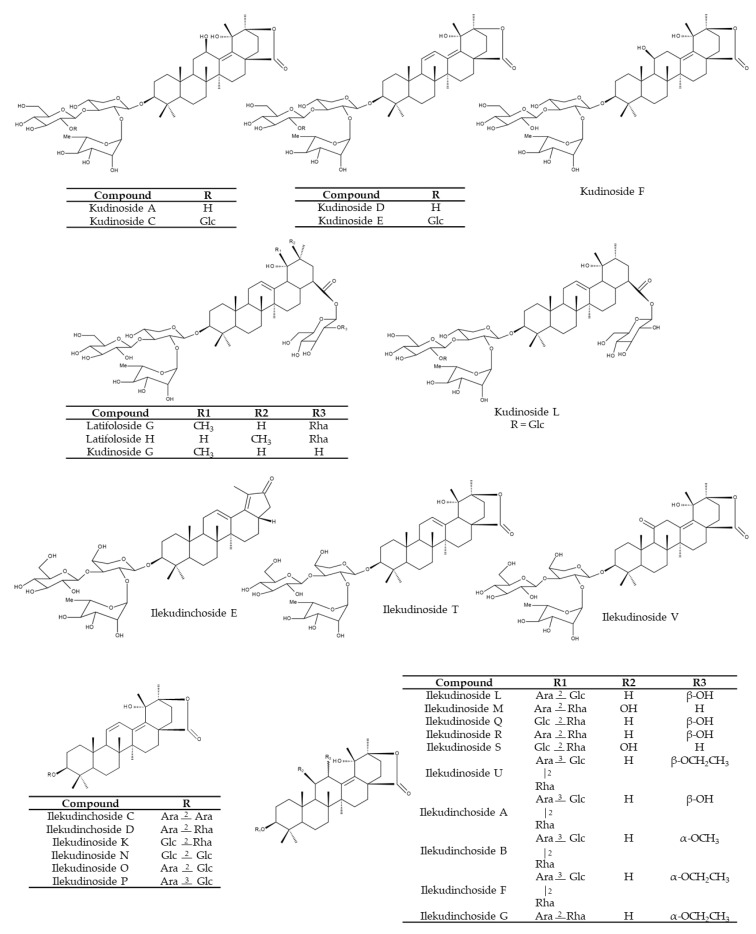
Chemical structures of the saponins in Kuding tea. Ara, arabinoside; Glc, glucoside; Rha, rhamnoside.

Triterpene saponins possessing ursane-type triterpenoids as aglycones are considered to be the most characteristic constituents of KT [1,22,27,28]. In addition, oleanane- and lupine-type triterpenoids and their glycosides are also present in KT [1]. A chemical analysis of 45 *Ilex* species indicated that *I. kudingcha* exhibited the highest content of saponins [13]. Comparing the content of five triterpenoid saponins (including α-, β- and γ-kudinlactones) tested in these 45 *Ilex* samples revealed some similarities within the same species but differences between the young and old leaves from the same species. Furthermore, differences among various species have been found [13]. Kudinosides C and A, both β-kudinlactones, are the main saponins present in *I. latifolia*, while kudinoside F (α-kudinlactone) and kudinoside D (γ-kudinlactone) were not found [13]. Feng et al. [29], in turn, determined 12 main saponins in *I. latifolia*, among them kudinosides D, A, H. Song et al. [5] identified 52 saponins in an aqueous extract of *I. kudingcha*, including the three major saponins: kudinosides A, D and F. The content of latifoloside G, kudinoside A and kudinoside G constitute such a large proportion (>80%), that these three molecules are suggested as suitable index compounds of total saponins in *I. kudingcha*, a relevant issue in quality control [15]. Over the years, researchers have isolated and identified a broad range of saponins, including kudinosides A-P [15,27,30,31,32], ilekudinosides A-T [15,30,33], latifolosides A-Q [15,28,30,31,33] and ilekudinchosides A-G and T-V [26,34,35,36] (Figure 5).

Ultrasonication with 75% (*v*/*v*) methanol for 30 min turned out to be a feasible extraction method to obtain saponins from KT [13].

### 3.2. Polyphenols

It has been shown that the total polyphenol content of six different Kudingcha genotypes from the genus *Ilex* was on average ~100 mg gallic acid equivalents (GAE)/g dry weight (DW), detected by a total phenol assay using the Folin–Ciocalteu reagent (FCR) [14]. The FCR-based assay is a colourimetric method that has become a routine procedure to determine total phenol content [37]. Zhang et al. [17] detected a total polyphenol content of ~190 mg GAE/g DW in *I. latifolia* [17], whereas Hu et al. [23] showed a total phenolic content of ~85 mg GAE/g DW in an *I. latifolia* ethanolic extract [23].

**Figure 6:**
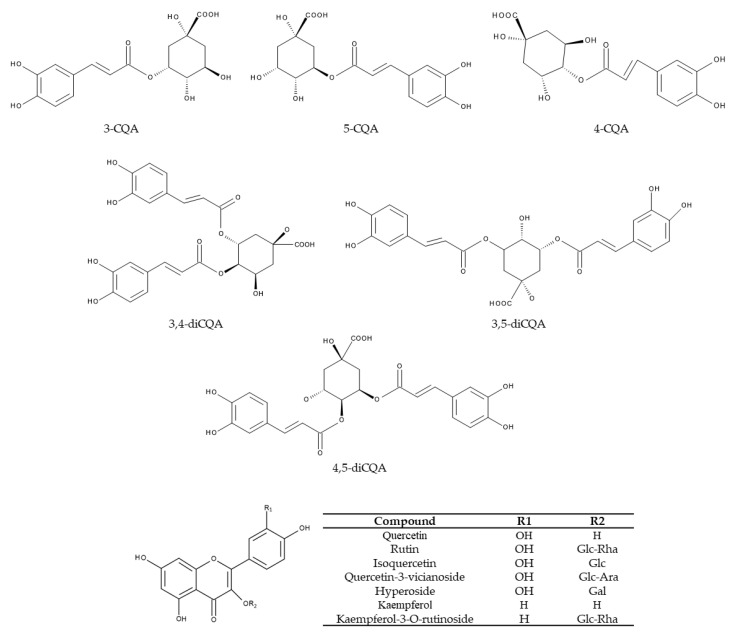
Chemical structures of the main polyphenols in Kuding tea. Ara, arabinoside; CQA, caffeoylquinic acid; Gal, galactose; Glc, glucoside; Rha, rhamnoside.

The top six compounds amongst the polyphenols were chlorogenic acid, neochlorogenic acid, cryptochlorogenic acid and isochlorogenic acids A, B and C (Table 1 and Figure 6) [14,15,19,21,38,39,40]. Structurally, chlorogenic acids consist of a caffeic acid moiety and a quinic acid moiety. There is nomenclature discrepancy regarding chlorogenic acid, since it is known as both 5-caffeoylquinic acid and 3-caffeoylquinic acid [41]. In this review, chlorogenic acid refers to 5-caffeoylquinic acid. By employing high-performance liquid chromatography (HPLC) analyses, Zhao et al. [39] detected chlorogenic acid, cryptochlorogenic acid and isochlorogenic acids A, B and C as the major polyphenols in KT. This was confirmed by liquid chromatography-mass spectrometry (LC-MS) analyses, where chlorogenic acids and their derivatives were the major polyphenols in *I. latifolia* Thunb [40]. In addition, isochlorogenic acids A, B and C were the three dominant polyphenolic compounds in *I. kudingcha* [38]. Accordingly, Yi et al. [15] proposed that chlorogenic acid, isochlorogenic acid A and isochlorogenic acid C could be used as index components for quality assessment because the content summation of these three phenolic acids accounted for >75% of the total phenolic acids. In fact, isochlorogenic acid A had the highest content (~29–52 mg/g) in seven out of eight batches of *I. kudingcha* C.J. Tseng collected from different geographical locations [21]. In the same study, three compounds (isochlorogenic acid A, isochlorogenic acid C and neochlorogenic acid) accounted for approximately 90% of the total phenolic content out of the six major chlorogenic acids [21]. Apart from these major phenolic acids, KT contains a large number of minor chlorogenic acids. This was demonstrated in a study on *I. kudingcha*, where 68 chlorogenic acids from 12 categories were identified by using a sensitive ultra-high-performance liquid chromatography method with diode array detection coupled to a linear ion trap-Orbitrap (UHPLC-DAD-LTQ-Orbitrap) [21].

In addition, some flavonoids have been found in KT (Figure 6). For instance, there are reports that rutin [14,15,20,38,40,43], hyperoside [38,40], kaempferol [20], isoquercetin [43], quercetin-3-vicianoside [43], kaempferol-3-O-rutinoside [14,43] and quercetin [20] have been discovered.

To concentrate phenolic substances from KTE, ethyl acetate extraction gave the best results followed by methanol, n-butanol, chloroform, water and hexane [19,20,23].

## 4. Health Benefits

**Figure 7:**
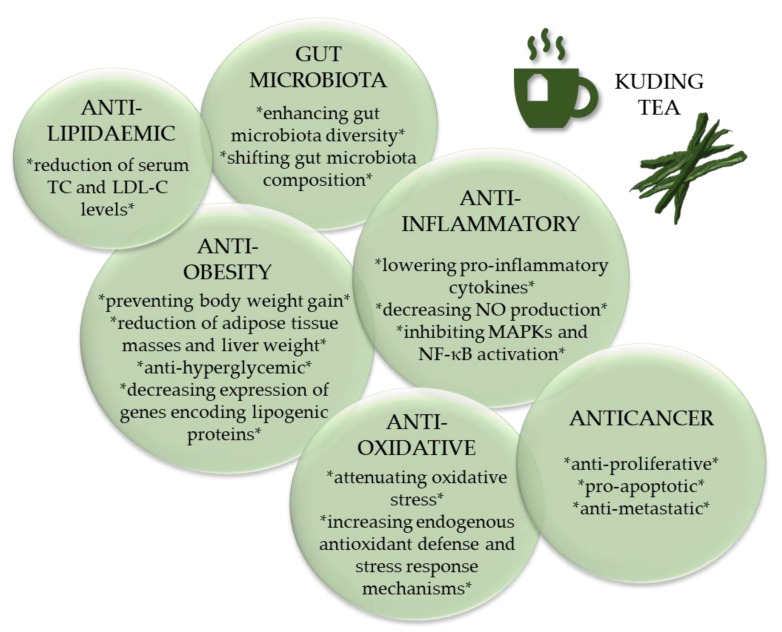
Bioactivity of Kuding tea and its constituents. LDL-C, low-density lipoprotein-cholesterol; MAPK, mitogen-activated protein kinase; NF-κB, nuclear factor-κB; NO, nitric oxide; TC, total cholesterol; * * bullet points.

Recently, several studies have focused on the antiobesity properties of KT. In particular, the effects of KT on lipid metabolism, body weight and blood glucose have gained increasing attention [3]. In this context, the intestinal microbiota has been identified as a putative novel target of KT. The research that will be discussed here includes only studies with extracts of *I. kudingcha* and *I. latifolia* as well as bioactive compounds such as polyphenols or saponins extracted from the genus *Ilex*. We will also address the relatively new field of KNP and KTE-γCD complexes and consider putative synergetic and antagonistic effects of different bioactive compounds present in KTE. The main biological processes that have been reported to be affected by KT and KT ingredients are summarised in Figure 7 and will be discussed with respect to human health benefits.

### 4.1. Antiobesity Activity

Several studies have demonstrated that KTE hinders adipocyte differentiation. An ethanol extract of KT (20 µg/mL) inhibited the differentiation of 3T3-L1 preadipocytes [3]. In detail, it has been found to impede the later stages of differentiation by lowering the mRNA expression of adipogenesis-related genes (peroxisome proliferator-activated receptor gamma, Pparγ; fatty acid-binding protein 4, Fabp4 and fatty acid synthase, Fasn). A water extract of *I. kudingcha* tested in the same study did not affect 3T3-L1 differentiation [3]. In contrast, Wu et al. [44] showed that an aqueous extract of *I. latifolia* (4 µg/mL) inhibited OP9 mouse stromal cell lipogenesis via Pparγ signalling, suggesting a different composition of bioactive components in the aqueous extracts of the two *Ilex* species.

In accordance with these cell culture data, a five-week feeding study in female C57BL/6 mice fed either a standard chow, a high-fat diet or a high-fat diet supplemented with an *I. kudingcha* 0.05% ethanol extract revealed that KTE blocks high-fat diet-induced body weight gain without suppressing food intake or inhibiting lipid absorption [3]. Similarly, the antiobesity potential of an aqueous extract of *I. latifolia* was confirmed by Wu et al. [44]. Male C57BL/6 mice received either a normal diet, a high-fat diet or a high-fat diet supplemented with 0.33% aqueous KTE for 14 weeks. Mice fed the KTE-supplemented diets gained less body weight than mice without KTE [44]. In both studies, it was suggested that KTE supplementation counteracted a high-fat diet-induced increase in white and brown fat mass in mice [3,44]. Furthermore, KTE treatment improved glucose homeostasis in high-fat diet-fed mice and prevented lipid accumulation in the liver [3,44]. Accordingly, the dietary *I. latifolia* extract was shown to protect against hepatic steatosis [44], and KTE improved metabolic disorders in obese mice [3].

By employing a transgenic reporter gene cell line, Fan et al. [3] found that the activation of liver X receptor-beta (LXRβ) by its agonist GW3965 was competitively inhibited by KTE. This might point to the underlying molecular mechanism, since LXRs are members of the nuclear receptor family of transcription factors and are important modulators of lipid and cholesterol homeostasis. In line with this, the expression levels of LXRβ target genes involved in fatty acid and cholesterol metabolism were significantly downregulated in KTE-treated mice [3]. In male C57BL/6 mice fed a high-fat diet, supplementation with 400 mg/kg/d aqueous *I. kudingcha* extract by intragastric gavage significantly alleviated body weight gain after an eight-week experimental period [4]. Furthermore, intragastric gavage of KTE reduced epididymal and perirenal fat depots as well as liver weight [4]. The gene expression level of LXRα showed no significant difference between the groups [4]. Oral administration of di-caffeoylquinic acids (diCQAs) derived from Kudingcha was reported to reduce liver weight, hepatic fat accumulation and adipose tissue masses in high-fat diet-fed mice. However, diCQA treatment had no significant effect on body weight [45]. Accumulating evidence has indicated that administration of aqueous or ethanolic KTE or its bioactive constituents decreased the expression of hepatic lipogenic genes such as Pparγ, sterol regulatory element-binding protein-1c (Srebp-1c) and Fasn compared to high-fat control mice and protected the mice from obesity by reducing lipogenesis [4,29,44,45] possibly via AMP-activated protein kinase (AMPK) pathway activation [29]. Moreover, diCQA treatment increased the expression of the hepatic lipid degradation-related genes carnitine palmitoyltransferase 1 (Cpt-1) and Pparα [45].

Bioactive components derived from KTE have also been tested for their antiobesity activity. Intragastric administration of total saponins from a water extract of KT exhibited liver-protective properties in high-fat diet-induced hyperlipidaemic mice via upregulation of the hepatic scavenger receptors steroid receptor RNA activator 1 (Sra1), scavenger receptor class B, member 1 (Scarb1) and the CD36 molecule (Cd36) [5]. Moreover, saponins from *I. latifolia* lowered body, liver and adipose tissue weights and suppressed the progression of hepatic steatosis in male mice fed a high-fat diet [29]. These data indicate that saponins represent key molecules for the biological activity of KTE. In alloxan-induced type 2 diabetic mice, four-week intragastric administration of 3.8 g of the active compounds of an aqueous KTE (phenolic acids, flavonoids and saponins) per kg per day (equivalent to 15 g *I. kudingcha*) resulted in a significantly lower body weight compared to the type 2 diabetic control mice. Furthermore, treatment with the KTE exhibited antihyperglycaemic effects in alloxan-induced type 2 diabetic mice [43]. In addition, *I. kudingcha* polysaccharides showed protective effects on body weight gain and liver steatosis induced by a high-fructose diet in mice [46].

Recently, novel technologies have been applied to enhance the bioavailability and bioactivity of the components of KT. Sprague-Dawley rats who received a high-fat diet supplemented with 50, 100 or 200 mg/kg KNP for four weeks showed reduced liver weight and adipose tissue mass compared to high-fat diet control rats [8]. The blank nanoparticle vehicle control group exhibited the same effects. However, liver and adipose tissue weights were significantly decreased in the high KNP administration group compared with the vehicle control [8]. The rats fed the high-fat control diet gained less weight than the rats fed a normal control diet. Nonetheless, medium and high KNP administration led to greater weight loss compared with the high-fat control diet [8]. The effects of dietary KTE-γCD in C57BL/6 mice fed a high-fat, high-fructose diet were recently examined [9]. Supplementation with KTE-γCD had no effect on body weight gain. However, the six-week experimental period led to increases in the absolute liver weight, hepatosomatic index and fat accumulation in hepatocytes [9], thereby presenting contrary reported liver-protective properties.

### 4.2. Antilipidaemic Activity

Several studies have demonstrated that KTE or its bioactive compounds improve plasma lipid parameters, especially when focusing on total cholesterol (TC) and low-density lipoprotein-cholesterol (LDL-C) levels [3,4,5,29,43,44,45,46,47]. Supplementation with 0.33% aqueous extract of *I. latifolia* for 14 weeks decreased TC and LDL-C levels in mice fed a high-fat diet [44]. Similarly, KTE treatment lowered the LDL-C plasma concentration in mice fed a high-fat diet supplemented with a 0.05% ethanolic extract of *I. kudingcha* for five weeks compared to high-fat diet control mice [3]. However, KTE showed no significant effects on blood lipid levels in male C57BL/6 mice fed a high-fat diet supplemented with 400 mg/kg/d aqueous *I. kudingcha* extract by intragastric gavage [4]. Conversely, mice fed a high-fat, high-fructose diet supplemented with KTE-γCD showed increased plasma TC levels [9].

Serum levels of TC, triglycerides and nonesterified fatty acids were improved by treatment with active components (phenolic acids, flavonoids and saponins) of an aqueous KTE in alloxan-induced type 2 diabetic mice compared to diabetic control mice [43]. Song et al. [5] attained hypolipidaemic effects in high-fat diet-induced hyperlipidaemic mice by treatment with total saponins from an aqueous *I. kudingcha* extract. The authors partly explained the results by the increased gene expression levels of the hepatic scavenger receptors steroid receptor RNA activator 1 (Sra1), scavenger receptor class B, member 1 (Scarb1) and Cd36 [5]. The hypolipidaemic activity was confirmed by Feng et al. for the saponins from *I. latifolia* [29] that lowered plasma triglycerides, TC and LDL-C in high-fat diet-fed mice. Furthermore, it was found that oral administration of diCQAs from *I. kudingcha* exhibited hypolipidaemic properties in high-fat diet mice [45]. Saponins and diCQAs from KT lowered serum total cholesterol and LDL-C levels, respectively [5,45].

Shi et al. [47] investigated the impact of saponins from *I. kudingcha* on serum lysoglycerophospholipids (Lyso-GPLs), potential biomarkers for hyperlipidaemia, in high-fat diet-induced hyperlipidaemic mice. Treatment with KT-derived saponins increased the content of Lyso-GPLs compared with mice receiving a high-fat control diet only. In addition, it has been shown that hyperlipidaemic mice exhibited decreased levels of Lyso-GPLs with a higher degree of unsaturation or a longer carbon fatty acyl chain. Administration of saponins from KT reversed this trend, confirming the positive metabolic change in serum Lyso-GPLs by KT saponins [47].

### 4.3. Regulation of the Intestinal Microbiota

Numerous secondary plant metabolites affect the gut microbiota. In line with this, KTE treatment was found to shape the intestinal microbiota community in feeding experiments. Dietary *I. kudingcha* increased the diversity of the gut microbiota and had considerable effects on gut microbiota composition in mice fed a high-fat diet. In detail, a high-fat diet negatively shifted the gut microbiota composition, and KTE treatment reversed this effect [4]. At the family level, KTE reduced the relative abundances of Erysipelotrichaceae and Coriobacteriaceae to the levels of normal diet-fed mice. From the 122 operational taxonomic units (OTUs) modified by a high-fat diet, KTE treatment reversed 30 [4].

The impact of *I. kudingcha* on gut microbiota was also investigated in mice with dextran sulfate sodium (DSS)-induced colitis [48]. The microbial composition in KTE-treated mice with DSS-induced colitis was similar to that in the healthy control group. Again, KTE had a significant effect on the relative abundances at the family level. Treatment with KTE reduced the fraction of Bacteroidaceae, Sutterellaceae, Clostridiaceae_1 and Erysipelotrichaceae. However, KTE had only a limited effect on the relative frequencies at the phylum level. Seventy-four OTUs were altered by DSS treatment, and KTE reversed 37 of these, indicating that KTE treatment alleviated microbiota dysbiosis [48].

It has been reported that diCQAs from *I. kudingcha* remained intact in stimulated saliva and gastric and pancreatic fluids. Only during the anaerobic fermentation with a human faecal slurry were the diCQAs hydrolysed to mono-CQAs and caffeic acid [49]. A strain of bacteria that hydrolyses diCQAs was identified as *Lactobacillus fermentum* LF-12 [50]. It was shown that diCQAs raised microbial diversity in vitro and in vivo [45,51]. The results from the *I. kudingcha* diCQAs feeding study in high-fat diet-fed C57BL/6 mice were partly in accordance with previous reports on the impact of *I. kudingcha* on gut microbiota. Administration of diCQAs reduced the relative abundances of Bacteroidaceae and Erysipelotrichaceae and reversed 40 OTUs towards the level of normal diet control mice [45].

### 4.4. Anticancer Properties

Recently, two papers reported the anticancer properties of *I. kudingcha* [6,7]. Both an aqueous [7] and an ethanolic [6] extract showed potent dose-dependent antiproliferative, proapoptotic and anti-inflammatory effects in TCA8113 human tongue carcinoma cells and MCF-7 human breast adeno-carcinoma cells [6,7]. Treatment with 200 µg/mL KTE inhibited the growth of MCF-7 cells up to ~80% [7]. KTE induced apoptosis in cancer cells via the BCL2-associated X protein (Bax)- and the B cell leukaemia/lymphoma 2 (Bcl-2)-dependent pathway accompanied by increased caspase-3 and caspase-9 gene expression levels [6,7].

A follow-up study by Zhao et al. [52] reported antiproliferative and apoptosis-inducing properties of polyphenols derived from *I. kudingcha* in the human buccal squamous cell carcinoma cell line BcaCD885 [52]. BcaCD885 cells were incubated with KT polyphenols for 48 h at concentrations of 25, 50 and 100 μg/mL. The highest KT polyphenol concentration exhibited an ~70% growth inhibition rate. Similar to the studies with KTE, treatment with KT polyphenols induced apoptosis in a dose-dependent manner. Again, proapoptotic properties were partly mediated via Bax and Bcl-2 [52]. Hence, polyphenols were suggested to be the main potential anticancer constituents of KT. Tea polyphenols have further been described to induce cell cycle arrest and apoptosis in human cancer cells [53]. This was confirmed by Hu et al. [23]. A 95% ethanol extract of *I. latifolia* and its ethyl acetate fraction at a concentration of 50 µg/mL exhibited the strongest antiproliferative effects against human cervical carcinoma HeLa cells (50 and 55%, respectively). Among the isolated compounds, two derivatives of caffeoylquinic acid (3,4-di-O-caffeoylquinic acid methyl ester and 3,5-di-O-caffeoylquinic acid methyl ester) showed the highest cytotoxicity against HeLa cells [23].

An ethanolic KTE was also effective in inhibiting metastasis in a dose-dependent manner in mice, demonstrating the preventive effects of KT in buccal mucosa cancer [6]. After showing in vitro that the antimetastatic activity of KTE was mediated through the downregulation of matrix metalloproteases (MMPs) and upregulation of tissue inhibitors of metalloproteases (TIMPs), Zhu et al. [6] determined metastasis inhibition activity of KTE in mice in vivo. The highest oral dose of KTE-solution (1600 mg/kg) in combination with KTE (400 mg/mL) topically applied to the buccal mucosa of the mice exhibited the strongest antimetastatic effect. In KTE-treated mice, the tumour volumes were smaller, and the buccal mucosa cancer degrees were weaker than those of control mice [6]. Zhao et al. [7] evaluated the development of lung metastasis in mice after KTE treatment. An aqueous KTE was subcutaneously injected at doses up to 1600 mg/kg. Two days after KTE injection, Zhao et al. [7] intravenously inoculated the mice with colon 26-M-3.1 cancer cells to study lung metastasis. After two weeks, all KTE-treated mice had fewer lung metastatic colonies compared to the control mice and were the most effective at the highest KTE concentration [7].

### 4.5. Anti-Inflammatory Activity

The anti-inflammatory properties of KTE were observed in feeding studies with rodents. High-fat diet-induced chronic inflammation was counteracted by KTE supplementation as indicated by the modulation of cytokine levels [4,44] and via the p38 mitogen-activated protein kinase (MAPK) [44] and the p65 nuclear factor-kappa B (NF-κB) signalling pathway [8,44]. *I. kudingcha* normalised serum proinflammatory cytokine levels in high-fat diet-fed mice to those of normal diet-fed mice [4]. In addition, *I. latifolia* increased murine serum levels of anti-inflammatory cytokines, including interleukin-4 (IL-4) and IL-10, under high-fat diet conditions [44].

The anti-inflammatory activity of an ethanolic *I. latifolia* extract (50–200 mg/kg) in transient, focal, ischaemia-induced brain damage using the MCAO/reperfusion model in rats was examined by Kim et al. [54]. Unlike the above-mentioned proapoptotic effects on cancer cells, KTE prevented apoptotic neuronal death thereby reducing ischaemic damage. Increased Bcl-2 levels and decreased Bax and caspase-3 levels were observed in KTE-treated rats. The inhibition of extracellular signalling-regulating kinases (ERK 1/2) and p38 MAPK phosphorylation by KTE was suggested to be the underlying mechanism that prevents apoptosis and, therefore, confers neuroprotection [54]. In C57BL/7 mice with DSS-induced colitis, KTE treatment by intragastric gavage notably inhibited the production of proinflammatory cytokines and alleviated typical symptoms and the colitic histological changes of inflammatory bowel diseases [48]. Zhao et al. [55] demonstrated the anti-inflammatory potential of KTE in a rodent-induced gastric injury model. In a two-week feeding study, injured rats were fed a control diet or the same diet supplemented with increasing doses of an aqueous extract of *I. kudingcha*. Serum levels of inflammatory cytokines, which are known to correlate with stomach injuries, were examined. The highest orally administered dose of 1000 mg/kg led to the strongest reduction of approximately 50% of both IL-6 and tumour necrosis factor-alpha (TNF-α) compared with the control group. In a follow-up study, the preventive effects of polyphenols from *I. kudingcha* on gastric injury were examined in a mouse model [39]. KT polyphenols were identified as potent components exhibiting anti-inflammatory activity. The highest KT polyphenol dose (100 mg/kg BW) lowered serum proinflammatory cytokine levels of IL-6, TNF-α and interferon-gamma (IFN-γ) to close to those of mice treated with the positive control ranitidine [39]. Polyphenols derived from *I. kudingcha* were also reported to inhibit skin damage caused by ultraviolet B-induced skin injury in SKH1 hairless mice. The antioxidative and anti-inflammatory properties of KT polyphenols plus the fact that they modulate skin proteins (MMPs, TIMPs) are probably responsible for their skin-protective properties [42].

The anti-inflammatory activities of polyphenols derived from *I. latifolia* was observed in murine RAW 264.7 macrophages. Release of the proinflammatory cytokines TNF-α, IL-1β and IL-6 was induced by oxidised low-density lipoproteins and LPS. Polyphenol treatment of macrophages inhibited cytokine release in a concentration-dependent manner [17,40]. KT polyphenols also decreased cyclooxygenase-2 (COX-2) levels [17] and nitric oxide (NO) production [17,23] by inhibiting inducible nitric oxide synthase (iNOS) in LPS-stimulated RAW 264.7 cells [17]. As an isolated compound, 3-CQA exhibited strong anti-inflammatory activity [23]. The anti-inflammatory effects of polyphenols from KT were proposed to be mediated through the inhibition of ERK and c-jun N-terminal kinase (JNK) MAPK phosphorylation and NF-κB activation [17,40].

The impact of KTE on inflammation in cancer cells has been described by Zhao et al. and Zhu et al. [6,7]. Aqueous and ethanolic extracts of *I. kudingcha* significantly regulated the expression of genes associated with inflammation in human breast and tongue carcinoma cells [6,7]. A concentration of 200 μg/mL aqueous KTE increased the gene expression of nuclear factor of kappa light polypeptide gene enhancer in B cell inhibitor, alpha (IκB-α) and decreased the gene expression of NF-κB in MCF-7 cells [7]. In TCA8113 human tongue carcinoma cells, treatment with 200 μg/mL ethanolic KTE significantly increased IκB-α levels and decreased the mRNA and protein levels of NF-κB accompanied by decreased COX-2 and iNOS mRNA and protein levels [6]. The synthesis of proinflammatory lipids such as prostaglandins is driven by COX enzymes, and the inducible enzyme COX-2 is upregulated in many cancers. Hence, COX-2 has been discussed as a putative anticancer target [56].

### 4.6. Antioxidative Activity

Hu et al. [23] evaluated the free radical scavenging activities of a crude ethanol extract and its four fractions (petroleum ether fraction, ethyl acetate fraction, butanol fraction and water fraction) from *I. latifolia* using the ferric reducing antioxidant power (FRAP) assay, 2,2-diphenyl-1-picrylhydrazyl (DPPH) radical scavenging assay and oxygen radical absorbance capacity (ORAC) assay in human cervical carcinoma HeLa cells. Based on these assays, the ethyl acetate fraction of *I. latifolia,* which contains the highest total phenolic content, exhibited the most potent free radical scavenging activity compared to the crude extract and the other three fractions [23]. More specifically, the level of free radical scavenging activity correlated with the total phenolic content observed by the phenol assay with FCR [23]. The results for *I. kudingcha* and its antioxidant activity support this relation [19]. Furthermore, this was confirmed by Zhu et al. [14], who showed that total phenolic acids from certain *Ilex* genotypes are highly associated with total phenolic content and free radical scavenging activity (ABTS, DPPH, FRAP) [14]. Moreover, all isolated phenolic acids from *I. kudingcha* showed free radical scavenging activities with diCQAs exhibiting higher free radical scavenging activities than monoCQAs [20]. To evaluate their antioxidative capacity, diCQAs isolated from *I. latifolia* and the ethanolic extract itself were applied in a glutamate- and hypoxia-induced neuronal cell death assay with cultured rat cortical neurons. Glutamate-induced neuronal cell death has been reported to be mediated via increased intracellular calcium (Ca^2+^) accompanied by the elevated generation of reactive oxygen species (ROS) and neuronal cell damage. DiCQAs and the ethanolic KT extract itself were found to counteract glutamate-induced neurotoxicity possibly due to their antioxidant and antiapoptotic properties (increased Bcl-2 and decreased Bax levels) [57].

It has been reported that high-fat or high-fructose diet intervention can lead to enhanced lipid peroxidation and higher oxidative stress in mouse livers [4,46]. In this context, treatment with KTE or some of its bioactive compounds, such as saponins or polysaccharides, has been reported to attenuate oxidative stress in rodent livers, combined with increased levels of endogenous antioxidative enzymes, including superoxide dismutase (SOD) and glutathione peroxidase (GSH-Px) [4,8,29,46,58]. In detail, 200 mg/kg polyphenols derived from Kudingcha led to increased activity of SOD, GSH-Px and glutathione (GSH) as well as decreased levels of free radical NO and MDA in the serum, liver and spleen of mice with D-galactose-induced oxidation. Accordingly, the mRNA and protein levels of the transcription factor nuclear factor erythroid-2-related factor (NRF2) and its target genes manganese and copper/zinc SOD (Mn-SOD, Cu/Zn-SOD), catalase (CAT), haem oxygenase-1 (HO-1), gamma-glutamylcysteine synthetase (γ-GCS) and NAD(P)H quinone oxidoreductase (NQO1) were higher in the livers and spleens of polyphenol-treated mice than those in control mice. Furthermore, Kudingcha polyphenols led to increased mRNA and protein levels of neuronal, endothelial and inducible nitric oxide synthase (nNOS, eNOS, iNOS) in mouse livers and spleens [59]. In addition, the antioxidative activity of Kudingcha polyphenols may contribute to the preventive effects reported for a colitis model in BALB/c mice. Polyphenol treatment reduced oxidative stress and increased the activity and mRNA levels of genes encoding antioxidant enzymes in colon tissues, including Mn-SOD, Cu/Zn-SOD, GSH-Px and CAT [60]. These findings indicate that KT-derived polyphenols protect against oxidative stress in vivo possibly via an indirect mode of action through modulating expression of genes encoding antioxidant enzymes [61] and not through their free radical scavenging activity per se like it has been reported in several in vitro studies [14,23,62]. Apart from polyphenols, polysaccharides isolated from *I. latifolia* were also found to reduce oxidative stress and improve the activity of SOD and GSH-Px in carbon tetrachloride-induced liver injury in mice [58]. Antioxidative activity of KNP was reported in high-fat diet-fed rats. Serum and liver MDA levels decreased after oral gavage of KNP for four weeks compared to high-fat diet control mice, while serum and liver SOD levels increased in KNP mice [8].

## 5. Safety Aspects of Large-Leaved Kudingcha

**Figure 8:**
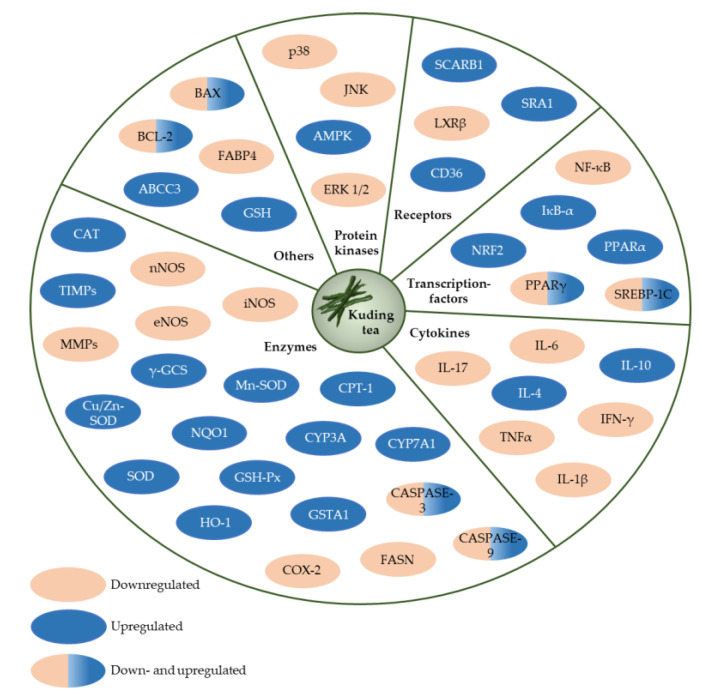
Potential molecular targets of large-leaved Kudingcha.These include, ABCC3, ATP-binding cassette, subfamily C, member 3; AMPK, AMP-activated protein kinase; BAX, BCL2-associated X protein; BCL-2, B cell leukemia/lymphoma 2; CASPASE-3, caspase 3; CASPASE-9, caspase 9; CAT, catalase; CD36, CD36 molecule; COX-2, cyclooxygenase-2; CPT-1, carnitine palmitoyltransferase 1; Cu/Zn-SOD, copper/zinc superoxide dismutase; CYP3A, cytochrome P450, family 3, subfamily a; CYP7A1, cytochrome P450, family 7, subfamily a, polypeptide 1; eNOS, endothelial nitric oxide synthase; ERK 1/2, extracellular signalling-regulating kinase 1/2; FABP4, fatty acid-binding protein 4; FASN, fatty acid synthase; GSH, glutathione; GSH-Px, glutathione peroxidase; GSTA1, glutathione S-transferase, alpha 1; HO-1, haem oxygenase 1; IFN-γ, interferon-gamma; IL-10, interleukin 10; IL-17, interleukin 17; IL-1β, interleukin 1 beta; IL-4, interleukin 4; IL-6, interleukin 6; iNOS, inducible nitric oxide synthase; IκB-α, nuclear factor of kappa light polypeptide gene enhancer in B cells inhibitor; JNK, c-Jun N-terminal kinase; LXRβ, liver X receptor-beta; MMPs, matrix metalloproteases; Mn-SOD, manganese superoxide dismutase; NF-κB, p65 nuclear factor-kappa B; nNOS, neuronal nitric oxide synthase; NQO1, NAD(P)H quinone oxidoreductase; NRF2, nuclear factor erythroid-2-related factor; p38, p38 mitogen-activated protein kinase; PPARα, peroxisome proliferator-activated receptor alpha; PPARγ, peroxisome proliferator-activated receptor gamma; SCARB1, scavenger receptor class B, member 1; SOD, superoxide dismutase; SRA1, steroid receptor RNA activator 1; SREBP-1C, sterol regulatory element-binding protein 1c; TIMPs, tissue inhibitors of metalloproteases; TNFα, tumor necrosis factor-alpha; γ-GCS, gamma-glutamylcysteine synthetase.

Traditional KT as a beverage has a long history of consumption and is generally considered to be safe [1]. A summary of the reported molecular targets in terms of protein kinases, receptors, transcription factors, cytokines and enzymes of large-leaved Kudingcha is given in Figure 8.

**Figure 9:**
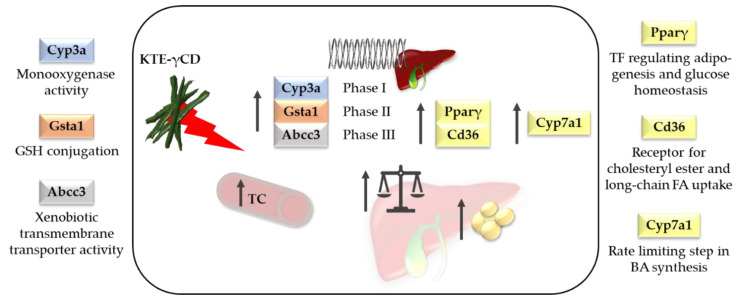
Safety aspects of a high dietary Kuding tea extract encapsulated in γ-cyclodextrin (KTE-γCD). KTE-γCD supplementation in mice induced hepatic xenobiotic-metabolising enzymes and increased liver mass accompanied by enhanced hepatic lipid accumulation. Abcc3, ATP-binding cassette, subfamily C, member 3; BA, bile acid; Cd36, CD36 molecule; Cyp3a, cytochrome P450, family 3, subfamily a; Cyp7a1, cytochrome P450, family 7, subfamily a, polypeptide 1; FA, fatty acid; GSH, glutathione; Gsta1, glutathione S-transferase, alpha 1; Pparγ, peroxisome proliferator-activated receptor gamma; TC, total cholesterol; TF, transcription factor [9].

However, owing to the increasing popularity of herbal medicine [63] and the development of high-dose formulations, the safety aspects of KTE as a nutraceutical or herbal drug need to be re-evaluated. It has been reported that KNP did not cause acute toxicity. In rats, doses up to 10,000 mg/kg given for 14 days by oral administration did not reach a death rate of 50%. Therefore, the median lethal dose (LD_50_) of KNP was set at >10,000 mg/kg [8]. However, in our recent study, we found that high-dose dietary KTE supplementation in the form of KTE encapsulated in γCD (KTE-γCD) induced fatty liver and increased hepatic xenobiotic-metabolising enzymes in mice after a six-week intervention [9]. Substantial induction of phase I and phase II enzymes and a phase III transporter of xenobiotic biotransformation was observed, indicating that the ingredients of KTE may interfere with xenobiotic/drug detoxification metabolism. In detail, induction of cytochrome P450, family 3, subfamily a (Cyp3a), glutathione S-transferase, alpha 1 (Gsta1) and ATP-binding cassette, subfamily C, member 3 (Abcc3) was reported. Furthermore, KTE-γCD-fed mice showed a higher liver mass accompanied by enhanced hepatic lipid accumulation and increased hepatic mRNA levels of the adipogenic transcription factor Pparγ and Cd36, a scavenger receptor for cholesteryl ester and long-chain fatty acid uptake [9]. A summary of the main adverse effects of KTE-γCD in mice from our study is given in Figure 9. Hepatotoxicity has been previously reported for certain herbal plants and phytoconstituents [63,64,65]. In this regard, it is of interest that the genes of phase I and II detoxification enzymes and phase III transporters are regulated by the redox-sensitive transcription factor Nrf2 [66]. The Nrf2-Kelch-like erythroid cell-derived protein with CNC homology [ECH]-associated protein 1 (Keap1) pathway is a key regulator of cell defence and survival through activating (amongst others) the cellular antioxidant response and xenobiotic-metabolising enzymes [66]. Zhao et al. [59] reported that polyphenols from Kudingcha activated the antioxidant responses of laboratory mice through the Nrf2 signalling pathway [59]. The pharmacological activation of the Nrf2 pathway has been discussed as a promising strategy to protect the liver [67]. Hence, according to Paracelsus’ central dictum “solely the dose determines that a thing is not a poison”, we suggest that moderate KTE supplementation may be cytoprotective and decrease reactive oxygen species and inflammation via an adequate Nrf2-mediated induction of cellular defence without eliciting the adverse effects observed for higher KTE concentrations.

## 6. Conclusions

This review summarises the classification, chemical composition, potential health benefits and safety aspects of the species *I. kudingcha* C.J. Tseng and *I. latifolia* Thunb from large-leaved Kudingcha. The main bioactive phytochemicals of KT are triterpenes, saponins and polyphenols. Taken together, KT exhibits antiobesity potential, which seems to be partly mediated by affecting intestinal microbiota. In addition, KT exhibits antioxidative and anti-inflammatory properties. Anti-proliferative, proapoptotic and antimetastatic effects of KT in cancer cells in vitro have been reported, which need to be confirmed in corresponding in vivo models. The physiological relevance of KT in humans remains rather unclear. The potential health benefits of KT and its constituents reviewed here are mainly derived on the basis of in vitro and animal studies. KT concentrations applied in cell culture studies are often much higher than those achieved due to dietary intake. Besides, in vitro studies often do not take the metabolic transformations of plant bioactives into account [62]. Furthermore, the route of KT administration should be also considered in studies employing animal models [68]. Finally, there is also evidence that high KTE supplementation can adversely affect liver metabolism [9].

## Figures and Tables

**Table 1:** Chlorogenic acid and its derivatives are the six major polyphenols in Kuding tea.

Polyphenols	Synonyms	Abbreviation	Content (mg/g)	References
neochlorogenic acid	3-caffeoylquinic acid	3-CQA	3.37 ^3^	[14,15,17,19,20,21,38,40,42]
chlorogenic acid	5-caffeoylquinic acid	5-CQA	105.49 ^1^ 79.71 ^2^ 30.51 ^3^	[14,15,17,19,20,21,38,39,40,42]
cryptochlorogenic acid	4-caffeoylquinic acid	4-CQA	20.69 ^1^ 16.02 ^2^ 2.59 ^3^	[14,15,20,21,38,39,40,42]
isochlorogenic acid B	3,4-di-caffeoylquinic acid	3,4-diCQA	163.65 ^1^ 52.53 ^2^ 4.51 ^3^	[15,19,20,21,38,39,42]
isochlorogenic acid A	3,5-di-caffeoylquinic acid	3,5-diCQA	70.33 ^1^ 120.03 ^2^ 52.18 ^3^	[14,15,19,20,21,38,39,42]
isochlorogenic acid C	4,5-di-caffeoylquinic acid	4,5-diCQA	129.49 ^1^ 96.42 ^2^ 30.32 ^3^	[14,15,19,20,21,38,39,42]

^1^ [39], ^2^ [42], ^3^ [21].

## References

[1] Li L., Xu L.J., Ma G.Z., Dong Y.M., Peng Y., Xiao P.G. (2013). The large-leaved Kudingcha (Ilex latifolia Thunb and Ilex kudingcha C.J. Tseng): A traditional Chinese tea with plentiful secondary metabolites and potential biological activities. J. Nat. Med..

[2] He Z.-D., Lau K.-M., But P.P.-H., Jiang R.-W., Dong H., Ma S.-C., Fung K.-P., Ye W.-C., Sun H.-D. (2003). Antioxidative glycosides from the leaves of Ligustrum robustum. J. Nat. Prod..

[3] Fan S., Zhang Y., Hu N., Sun Q., Ding X., Li G., Zheng B., Gu M., Huang F., Sun Y.-Q. (2012). Extract of Kuding tea prevents high-fat diet-induced metabolic disorders in C57BL/6 mice via liver X receptor (LXR) β antagonism. PLoS ONE.

[4] Chen G., Xie M., Dai Z., Wan P., Ye H., Zeng X., Sun Y. (2018). Kudingcha and Fuzhuan brick tea prevent obesity and modulate gut microbiota in high-fat diet fed mice. Mol. Nutr. Food Res..

[5] Song C., Yu Q., Li X., Jin S., Li S., Zhang Y., Jia S., Chen C., Xiang Y., Jiang H. (2016). the Hypolipidemic effect of Total Saponins from Kuding Tea in High-Fat Diet-Induced Hyperlipidemic Mice and Its Composition Characterized by UPLC-QTOF-MS/MS. J. Food Sci..

[6] Zhu K., Li G., Sun P., Wang R., Qian Y., Zhao X. (2014). In vitro and in vivo anti-cancer activities of Kuding tea (Ilex kudingcha C.J. Tseng) against oral cancer. Exp. Ther. Med..

[7] Zhao X., Wang Q., Qian Y., Song J.-L. (2013). Ilex kudingcha C.J. Tseng (Kudingcha) has in vitro anticancer activities in MCF-7 human breast adenocarcinoma cells and exerts anti-metastatic effects in vivo. Oncol. Lett..

[8] Zhang H., Zou X., Huang Q., Zhong X., Huang Z. (2018). Effects of Kudingcha nanoparticles in hyperlipidaemic rats induced by a high fat diet. Cell. Physiol. Biochem..

[9] Wüpper S., Fischer A., Lüersen K., Lucius R., Okamoto H., Ishida Y., Terao K., Rimbach G. (2019). High dietary Kuding tea extract supplementation induces hepatic xenobiotic-metabolizing enzymes-A 6-week feeding study in mice. Nutrients.

[10] Van den Berg S.J.P.L., Serra-Majem L., Coppens P., Rietjens I.M.C.M. (2011). Safety assessment of plant food supplements (PFS). Food Funct..

[11] Gan R.-Y., Zhang D., Wang M., Corke H. (2018). Health benefits of bioactive compounds from the genus ilex, a source of traditional caffeinated beverages. Nutrients.

[12] Hao D., Gu X., Xiao P., Liang Z., Xu L., Peng Y. (2013). Research progress in the phytochemistry and biology of Ilex pharmaceutical resources. Acta Pharm. Sin. B.

[13] Li L., Peng Y., Ma G., He C., Feng Y., Lei Q., Xiao P. (2012). Quantitative analysis of five kudinosides in the large-leaved Kudingcha and related species from the genus Ilex by UPLC-ELSD. Phytochem. Anal..

[14] Zhu F., Cai Y.-Z., Sun M., Ke J., Lu D., Corke H. (2009). Comparison of major phenolic constituents and in vitro antioxidant activity of diverse Kudingcha genotypes from Ilex kudingcha, Ilex cornuta, and Ligustrum robustum. J. Agric. Food Chem..

[15] Yi H., Zhou J., Shang X., Zhao Z., Peng Q., Zhu M., Zhu C., Lin C., Liu Q., Liao Q. (2018). Multi-component analysis of Ilex Kudingcha C.J. Tseng by a single marker quantification method and chemometric discrimination of HPLC fingerprints. Molecules.

[16] Wang C.-Q., Li M.-M., Zhang W., Wang L., Fan C.-L., Feng R.-B., Zhang X.-Q., Ye W.-C. (2015). Four new triterpenes and triterpene glycosides from the leaves of Ilex latifolia and their inhibitory activity on triglyceride accumulation. Fitoterapia.

[17] Zhang T.-T., Hu T., Jiang J.-G., Zhao J.-W., Zhu W. (2018). Antioxidant and anti-inflammatory effects of polyphenols extracted from Ilex latifolia Thunb. RSC Adv..

[18] Zhou J., Yi H., Zhao Z.-X., Shang X.-Y., Zhu M.-J., Kuang G.-J., Zhu C.-C., Zhang L. (2018). Simultaneous qualitative and quantitative evaluation of Ilex kudingcha C. J. tseng by using UPLC and UHPLC-qTOF-MS/MS. J. Pharm. Biomed. Anal..

[19] Liu L., Sun Y., Laura T., Liang X., Ye H., Zeng X. (2009). Determination of polyphenolic content and antioxidant activity of kudingcha made from Ilex kudingcha C.J. Tseng. Food Chem..

[20] Thuong P.T., Su N.D., Ngoc T.M., Hung T.M., Dang N.H., Thuan N.D., Bae K., Oh W.K. (2009). Antioxidant activity and principles of Vietnam bitter tea Ilex kudingcha. Food Chem..

[21] Che Y., Wang Z., Zhu Z., Ma Y., Zhang Y., Gu W., Zhang J., Rao G. (2016). Simultaneous qualitation and quantitation of chlorogenic acids in Kuding tea using ultra-high-performance liquid chromatography-diode array detection coupled with linear ion trap-orbitrap mass spectrometer. Molecules.

[22] Ouyang M.-A., Yang C.-R., Chen Z.-L., Wang H.-Q. (1996). Triterpenes and triterpenoid glycosides from the leaves of Ilex kudincha. Phytochemistry.

[23] Hu T., He X.-W., Jiang J.-G. (2014). Functional analyses on antioxidant, anti-inflammatory, and antiproliferative effects of extracts and compounds from Ilex latifolia Thunb., a Chinese bitter tea. J. Agric. Food Chem..

[24] Liu H., Liu C., Yang X., Zeng S., Xiong Y., Xu W. (2008). Solid-phase extraction of ursolic acid from herb using beta-cyclodextrin-based molecularly imprinted microspheres. J. Sep. Sci..

[25] Nishimura K., Fukuda T., Miyase T., Noguchi H., Chen X.M. (1999). Activity-guided isolation of triterpenoid acyl CoA cholesteryl acyl transferase (ACAT) inhibitors from Ilex kudincha. J. Nat. Prod..

[26] Zuo W.-J., Dai H.-F., Chen J., Chen H.-Q., Zhao Y.-X., Mei W.-L., Li X., Wang J.-H. (2011). Triterpenes and triterpenoid saponins from the leaves of Ilex kudincha. Planta Med..

[27] Ouyang M.-A., Wang H.-Q., Chen Z.-L., Yang C.-R. (1996). Triterpenoid glycosides from Ilex kudincha. Phytochemistry.

[28] Ouyang M.-A., Han-Qing W., Yu-Qing L., Chong-Ren Y. (1997). Triterpenoid saponins from the leaves of Ilex latifolia. Phytochemistry.

[29] Feng R.-B., Fan C.-L., Liu Q., Liu Z., Zhang W., Li Y.-L., Tang W., Wang Y., Li M.-M., Ye W.-C. (2015). Crude triterpenoid saponins from Ilex latifolia (Da Ye Dong Qing) ameliorate lipid accumulation by inhibiting SREBP expression via activation of AMPK in a non-alcoholic fatty liver disease model. Chin. Med..

[30] Tang L., Jiang Y., Chang H.-T., Zhao M.-B., Tu P.-F., Cui J.-R., Wang R.-Q. (2005). Triterpene saponins from the leaves of Ilex kudingcha. J. Nat. Prod..

[31] Ouyang M.-A. (2001). Glycosides from the Leaves of Ilex latifolia. Chin. J. Chem..

[32] Ouyang M.A., Yang C.R., Wu Z.J. (2001). Triterpenoid saponins from the leaves of Ilex kudincha. J. Asian Nat. Prod. Res..

[33] Nishimura K., Miyase T., Noguchi H. (1999). Triterpenoid saponins from Ilex kudincha. J. Nat. Prod..

[34] Zuo W.-J., Dai H.-F., Zeng Y.-B., Wang H., Chen H.-Q., Wang J.-H. (2012). Two new triterpenoid saponins from the leaves of Ilex kudingcha. J. Asian Nat. Prod. Res..

[35] Zuo W., Wang Q., Li W., Sha Y., Li X., Wang J. (2012). Structure elucidation and NMR assignments of an unusual triterpene saponin derivative from Ilex kudincha. Magn. Reson. Chem..

[36] Tang L., Jiang Y., Tian X.-M., Zhou S.-X., Tu P.-F. (2009). Triterpene saponins from the leaves of Ilex kudingcha. J. Asian Nat. Prod. Res..

[37] Huang D., Ou B., Prior R.L. (2005). The chemistry behind antioxidant capacity assays. J. Agric. Food Chem..

[38] Li H., Wang L., Luo Y. (2018). Composition analysis by UPLC-PDA-ESI (-)-HRMS and antioxidant activity using Saccharomyces cerevisiae model of herbal teas and green teas from Hainan. Molecules.

[39] Zhao X., Sun P., Li G., Yi R., Qian Y., Park K.-Y. (2018). Polyphenols in Kuding tea help prevent HCl/ethanol-induced gastric injury in mice. Food Funct..

[40] Zhang T.-T., Zheng C.-Y., Hu T., Jiang J.-G., Zhao J.-W., Zhu W. (2018). Polyphenols from Ilex latifolia Thunb. (a Chinese bitter tea) exert anti-atherosclerotic activity through suppressing NF-κB activation and phosphorylation of ERK1/2 in macrophages. Medchemcomm.

[41] Santana-Gálvez J., Cisneros-Zevallos L., Jacobo-Velázquez D.A. (2017). Chlorogenic Acid: Recent advances on its dual role as a food additive and a nutraceutical against metabolic syndrome. Molecules.

[42] Yi R., Zhang J., Sun P., Qian Y., Zhao X. (2019). Protective effects of Kuding tea (Ilex kudingcha C. J. Tseng) polyphenols on UVB-Induced skin aging in SKH1 hairless mice. Molecules.

[43] Song C., Xie C., Zhou Z., Yu S., Fang N. (2012). Antidiabetic effect of an active components group from Ilex kudingcha and its chemical composition. Evid. Based Complement. Alternat. Med..

[44] Wu H., Chen Y.-L., Yu Y., Zang J., Wu Y., He Z. (2017). Ilex latifolia Thunb protects mice from HFD-induced body weight gain. Sci. Rep..

[45] Xie M., Chen G., Wan P., Dai Z., Zeng X., Sun Y. (2019). Effects of Dicaffeoylquinic acids from Ilex kudingcha on lipid metabolism and intestinal microbiota in high-fat-diet-fed mice. J. Agric. Food Chem..

[46] Zhai X., Ren D., Luo Y., Hu Y., Yang X. (2017). Chemical characteristics of an Ilex Kuding tea polysaccharide and its protective effects against high fructose-induced liver injury and vascular endothelial dysfunction in mice. Food Funct..

[47] Shi Q., Jin S., Xiang X., Tian J., Huang R., Li S., Chen C., Xu H., Song C. (2019). The metabolic change in serum lysoglycerophospholipids intervened by triterpenoid saponins from Kuding tea on hyperlipidemic mice. Food Funct..

[48] Wan P., Peng Y., Chen G., Xie M., Dai Z., Huang K., Dong W., Zeng X., Sun Y. (2019). Modulation of gut microbiota by Ilex kudingcha improves dextran sulfate sodium-induced colitis. Food Res. Int..

[49] Xie M., Chen G., Hu B., Zhou L., Ou S., Zeng X., Sun Y. (2016). Hydrolysis of Dicaffeoylquinic acids from Ilex kudingcha happens in the colon by intestinal microbiota. J. Agric. Food Chem..

[50] Liu Y., Xie M., Wan P., Chen G., Chen C., Chen D., Yu S., Zeng X., Sun Y. (2020). Purification, characterization and molecular cloning of a dicaffeoylquinic acid-hydrolyzing esterase from human-derived Lactobacillus fermentum LF-12. Food Funct..

[51] Xie M., Chen G., Wan P., Dai Z., Hu B., Chen L., Ou S., Zeng X., Sun Y. (2017). Modulating effects of Dicaffeoylquinic acids from Ilex kudingcha on intestinal microecology in vitro. J. Agric. Food Chem..

[52] Zhao X., Pang L., Li J., Song J.-L., Qiu L.-H. (2014). Apoptosis inducing effects of Kuding tea polyphenols in human buccal squamous cell carcinoma cell line BcaCD885. Nutrients.

[53] Mukhtar H., Ahmad N. (2000). Tea polyphenols: Prevention of cancer and optimizing health. Am. J. Clin. Nutr..

[54] Kim J.Y., Jeong H.Y., Lee H.K., Yoo J.K., Bae K., Seong Y.H. (2011). Protective effect of Ilex latifolia, a major component of “kudingcha”, against transient focal ischemia-induced neuronal damage in rats. J. Ethnopharmacol..

[55] Zhao X., Wang Q., Qian Y., Song J.-L. (2013). Ilex kudingcha C.J. Tseng (Kudingcha) prevents HCl/ethanol-induced gastric injury in Sprague-Dawley rats. Mol. Med. Rep..

[56] Menter D.G., Schilsky R.L., DuBois R.N. (2010). Cyclooxygenase-2 and cancer treatment: Understanding the risk should be worth the reward. Clin. Cancer Res..

[57] Kim J.Y., Lee H.K., Hwang B.Y., Kim S., Yoo J.K., Seong Y.H. (2012). Neuroprotection of Ilex latifolia and caffeoylquinic acid derivatives against excitotoxic and hypoxic damage of cultured rat cortical neurons. Arch. Pharm. Res..

[58] Fan J., Wu Z., Zhao T., Sun Y., Ye H., Xu R., Zeng X. (2014). Characterization, antioxidant and hepatoprotective activities of polysaccharides from Ilex latifolia Thunb. Carbohydr. Polym..

[59] Zhao X., Song J.-L., Yi R., Li G., Sun P., Park K.-Y., Suo H. (2018). Comparison of antioxidative effects of insect tea and its raw tea (Kuding tea) polyphenols in kunming mice. Molecules.

[60] Long X., Pan Y., Zhao X. (2018). Prophylactic effect of Kudingcha polyphenols on oxazolone induced colitis through its antioxidant capacities. Food Sci. Hum. Wellness.

[61] Finley J.W., Kong A.-N., Hintze K.J., Jeffery E.H., Ji L.L., Lei X.G. (2011). Antioxidants in foods: State of the science important to the food industry. J. Agric. Food Chem..

[62] Serreli G., Deiana M. (2019). In vivo formed metabolites of polyphenols and their biological efficacy. Food Funct..

[63] Bedi O., Bijjem K.R.V., Kumar P., Gauttam V. (2016). Herbal induced hepatoprotection and hepatotoxicity: A critical review. Indian J. Physiol. Pharmacol..

[64] Oketch-Rabah H.A., Roe A.L., Rider C.V., Bonkovsky H.L., Giancaspro G.I., Navarro V., Paine M.F., Betz J.M., Marles R.J., Casper S. (2020). United States pharmacopeia (USP) comprehensive review of the hepatotoxicity of green tea extracts. Toxicol. Rep..

[65] Navarro V.J., Khan I., Björnsson E., Seeff L.B., Serrano J., Hoofnagle J.H. (2017). Liver injury from herbal and dietary supplements. Hepatology.

[66] Jaramillo M.C., Zhang D.D. (2013). The emerging role of the Nrf2-Keap1 signaling pathway in cancer. Genes Dev..

[67] Xu D., Xu M., Jeong S., Qian Y., Wu H., Xia Q., Kong X. (2018). The role of Nrf2 in liver disease: Novel molecular mechanisms and therapeutic approaches. Front. Pharmacol..

[68] Mena P., Del Rio D. (2018). Gold standards for realistic (Poly)phenol research. J. Agric. Food Chem..

